# Integrating oxidative ecology into conservation physiology

**DOI:** 10.1093/conphys/cot004

**Published:** 2013-04-05

**Authors:** Michaël Beaulieu, Anne-Mathilde Thierry, Daniel González-Acuña, Michael J. Polito

**Affiliations:** 1 Faculty of Biology, Department of Evolutionary Biology and Animal Ecology, University of Freiburg, Hauptstraße 1, 79104 Freiburg, Germany; 2 Université de Strasbourg, IPHC, 23 rue Becquerel, 67087 Strasbourg, France; 3 Facultad de Ciencias Veterinarias, Universidad de Concepción, Casilla 537, Chillán, Chile; 4 Woods Hole Oceanographic Institution, 266 Woods Hole Road MS 50, Woods Hole, MA 02543, USA

**Keywords:** Antarctica, demography, oxidative balance, penguins, population decline, seabirds

## Abstract

Ecologists have recently shown great interest in using physiological markers as indicators of the health of animal populations. In this context, the measurement of markers of oxidative balance, such as antioxidant defences and oxidative damage, may be a valuable tool. Indeed, at the individual level, antioxidant defences are positively associated with fertility and survival probability, while elevated oxidative damage during reproduction or growth may negatively affect recruitment and survival. Therefore, variation in oxidative balance is likely to influence demographic processes. This suggests that conservationists may be able to use oxidative markers to monitor population health. Yet, the connection between these markers and demographic parameters first needs to be established. We present here preliminary results obtained in colonies of breeding Gentoo (*Pygoscelis papua*) and Adélie penguins (*Pygoscelis adeliae*), showing that antioxidant defences strongly reflect population trends. However, population trend was not related to oxidative damage. This suggests that in the context of the emerging field of conservation physiology, antioxidant defences may represent a key parameter to monitor population health. We therefore exhort other research teams to assess the generality of this finding in other biological models, especially in species of conservation concern.

## Abstract (precis)

Given that oxidative balance acts on fitness components, its measurement may be valuable to conservationists to assess population health. We show that antioxidant defences reflect population trends in penguin colonies. These preliminary results suggest that oxidative balance could be used to assess the health of animal populations in their habitat.

## Why may markers of oxidative balance be worth examining in the context of conservation physiology?

Conservationists need accurate tools to evaluate the health of animal populations in their natural habitat. Towards this end, they have recently started to use physiological markers as indicators of population health (Wikelski and Cooke, 2006). So far, most research in conservation physiology has concentrated on endocrine and immunological parameters in response to environmental perturbations (Stevenson *et al.*, 2005). In comparison to endocrine and immunological ecology, oxidative ecology is a relatively recent research field, and has not yet been considered with respect to conservation issues. Yet, the measurement of oxidative markers may be valuable in view of the ubiquity of oxidative processes in biological systems and of their effects on individuals’ fitness.

Excesses of reactive oxygen species (ROS) relative to antioxidant defences result in the production of oxidative damage [because reactive nitrogen species are thought not to be as damaging as ROS (Monaghan *et al.*, 2009), we will not consider them in the present manuscript]. For instance, this may happen when organisms experience acute or prolonged physical activity (Costantini *et al.*, 2008; Fletcher *et al.*, 2012), when they reproduce (Alonso-Álvarez *et al.*, 2010; Christe *et al.*, 2012; Van de Crommenacker *et al.*, 2012), or when they are exposed to anthropogenic pro-oxidative agents (Bonisoli-Alquati *et al.*, 2010; Koivula and Eeva, 2010). High levels of oxidative damage can lead to accelerated cell ageing, thereby decreasing fertility and survival probability (Haenold *et al.*, 2005; Monaghan *et al.*, 2009). Accordingly, individuals with enhanced antioxidant defences or reduced oxidative damage have better fertility and survival (Bize *et al.*, 2008; Freeman-Gallant *et al.*, 2011; Saino *et al.*, 2011; Losdat *et al.*, 2012; Noguera *et al.*, 2012). Moreover, the intrinsic oxidative balance of organisms interacts with the oxidative constraints (e.g. radiation, temperature, and prey availability) imposed by environmental conditions (Beaulieu *et al.*, 2010). Such interaction may lead populations with low antioxidant defences to decrease when faced with pro-oxidative environmental conditions (Møller and Mousseau, 2007). This raises the hypothesis that oxidative balance and demographic processes may be interrelated, and that oxidative markers, reflecting demographic trends, may be used by conservationists as indicators of population health. Yet, the connection between oxidative balance and demographic parameters still needs to be established.

It may be initially assumed that increased levels of physiological markers classically associated with deteriorated fitness parameters at the individual level would reflect poor population health. However, such an assumption does not necessarily hold true. For instance, even if increased corticosterone levels predict decreased reproductive performance in common murres (*Uria lumvia*), corticosterone levels do not differ significantly between increasing and declining colonies, even when population trends differ drastically (Kitaysky *et al.*, 2007). Consequently, even if corticosterone levels may act on fitness components, their measurement appears inappropriate to estimate the health of populations of common murres. This example emphasizes the necessity to assess the relationship between physiological markers and demographic indices directly, before being able to use them as indicators of population health. The examination of oxidative balance in populations of known contrasting trends may therefore be a useful first step to assess the validity of the relationship between oxidative balance and population health.

## Examination of oxidative balance in populations of *Pygoscelis* penguins with contrasting population trends

According to the International Union for Conservation of Nature (IUCN), two of the three currently existing *Pygoscelis* species, namely the Gentoo penguin (*Pygoscelis papua*) and the Adélie penguin (*Pygoscelis adeliae*), are near threatened (IUCN, 2012). However, their status varies greatly between populations, which show contrasting demographic trends depending both on species and region. Indeed, over the few last decades, populations of Gentoo penguins have been stable or increasing around the Antarctic Peninsula, while populations of Adélie penguins have been decreasing (Hinke *et al.*, 2007). Conversely, in other regions of Antarctica, populations of Adélie penguins have been increasing (Table 1). The high dependence of Adélie penguins on decreasing stocks of Antarctic krill (*Euphausia superba*) around the Antarctic Peninsula is likely to be responsible for their decline in this region (Trivelpiece *et al.*, 1987; Hinke *et al.*, 2007). Compared with other populations, Adélie penguins from the Antarctic Peninsula may therefore have to intensify their foraging effort to feed. This may result in lower antioxidant defences and higher oxidative damage, as observed in birds increasing their flying effort (Costantini *et al.*, 2008). Moreover, they may be limited in their ability to invest in antioxidant defences, because their feeding requirements may not be entirely satisfied, and their endogenous resources may be limited. Finally, low consumption of krill, rich in antioxidants (Tou *et al.*, 2007), may result in low antioxidant defences and high oxidative damage (Beaulieu *et al.*, 2010). These potential changes in oxidative balance may contribute to explaining the negative impact of krill depletion on demographic parameters in Adélie penguins (Nicol *et al.*, 2008; Tierney *et al.*, 2009; Trivelpiece *et al.*, 2011).

**Table 1:** Average annual population changes of Gentoo and Adélie penguins from the Antarctic Peninsula (Ardley Island, Gabriel González Videla, and Admiralty Bay) and Adélie Land (Dumont D'Urville)

Species	Population	Coordinates	Annual population change (%)	No. of samples
Gentoo	Ardley Island	62°13'S, 58°54'W	+4.7 (1973–2005)^a^	14
Gentoo	Gabriel González Videla	64°49'S, 62°52'W	+3.8 (1986–1997)^b^	25
Adélie	Dumont d'Urville	66°40'S, 140°01'E	+1.8 (1984–2003)^c^	23
Gentoo	Admiralty Bay	62°11'S, 58°26'W	0 (1977–2005)^d^	20
Adélie	Admiralty Bay	62°11'S, 58°26'W	−2.3 (1979–2009)^e^	22

The time scale during which population changes were calculated is indicated in parentheses. The population trend of Gentoo penguins from Gabriel González Videla was approximated from that of the 10 km distant colony on Goudier Island (Cobley and Shears, 1999). The co-ordinates and the number of penguins from each population included in our study are also indicated. ^a^Antarctic Treaty Consultative Meeting (2010); ^b^Cobley and Shears (1999); ^c^Jenouvrier *et al.* (2005); ^d^Hinke *et al.* (2007); and ^e^Trivelpiece *et al.* (2011).

As Adélie penguins from the Antarctic Peninsula exhibit decreasing population trends, they are expected to have lower antioxidant capacity and higher oxidative damage than (i) Gentoo penguins from the same region, and (ii) Adélie penguins from other Antarctic regions. In order to assess this prediction, we examined the oxidative balance of breeding Gentoo and Adélie penguins from three different regions of the Antarctic Peninsula (Ardley Island, Gabriel González Videla, and Admiralty Bay) and from Adélie Land (Dumont d'Urville). For each colony, the published results of long-term population-monitoring studies provided us with mean annual population changes (Table 1). Oxidative balance was measured in plasma samples collected during the chick-rearing period of the austral summer 2010–2011, by using the OXY-adsorbent tests (Diacron International, Grosseto, Italy), which measures total antioxidant capacity, and the d-ROM test (Diacron International, Grosseto, Italy), which measures hydroperoxide, resulting from the attack of ROS on organic substrates (and therefore reflecting oxidative damage; for details on the procedure, see Beaulieu *et al.*, 2010).

**Figure 1:**
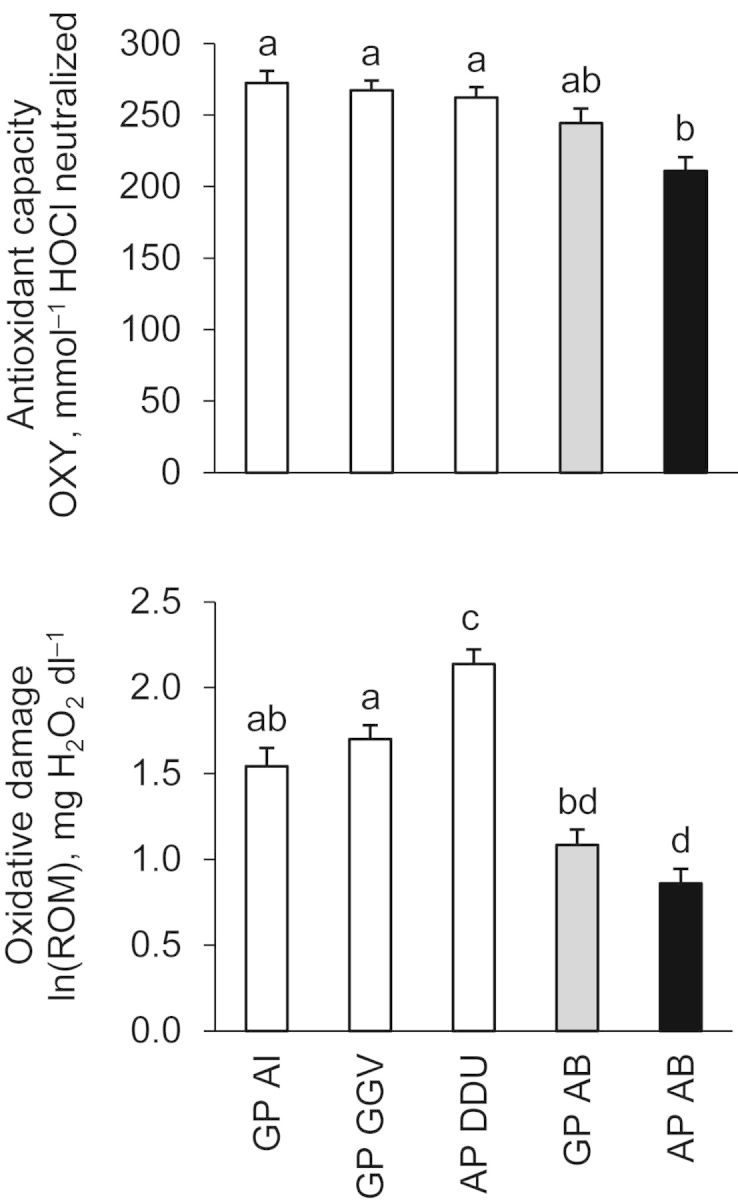
Plasma antioxidant capacity and oxidative damage in Gentoo (GP) and Adélie (AP) penguins from Ardley Island (AI), Gabriel González Videla (GGV), Dumont d'Urville (DDU), and Admiralty Bay (AB). Different letters indicate significant differences between colonies, as calculated by Bonferonni tests following Model 1 (Table 2). Increasing colonies are represented in white, stable in grey, and decreasing in black. Results are presented as means ± SEM.

**Figure 2:**
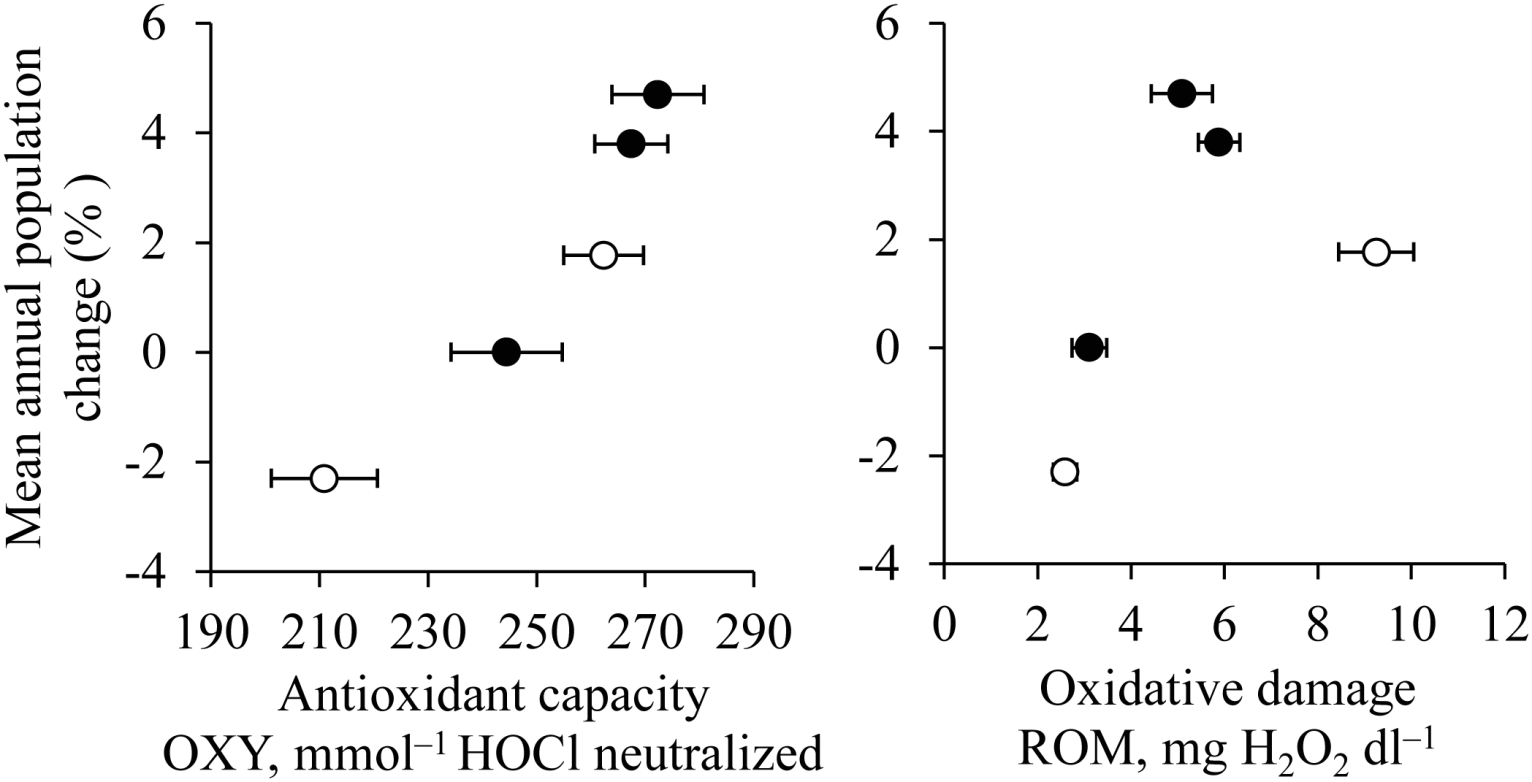
Relationships between mean annual population change and antioxidant capacity (means ± SEM, left), and between mean annual population change and oxidative damage (means ± SEM, right) in populations of Gentoo (filled circles) and Adélie penguins (open circles).

In agreement with our initial hypothesis, penguins from increasing populations had higher antioxidant capacity than penguins from decreasing populations (Fig. 1). This was true when considering each penguin colony independently (Table 2, Model 1; Fig. 2) or irrespective of species, location (Table 2, Model 2), and levels of oxidative damage (Table 2, Model 3). This positive relationship between antioxidant capacity and population trend may be related to the adaptive advantages conferred by higher antioxidant defences, such as enhanced fertility and survival, as already observed in other bird species (Bize *et al.*, 2008; Saino *et al.*, 2011; Losdat *et al.*, 2012). Moreover, penguins from declining populations may be unable to keep antioxidant defences as high as penguins from increasing colonies, because they are energetically limited and cannot invest effectively in antioxidant defences (Fletcher *et al.*, in press).

**Table 2:** Results of the statistical models used to assess the relationship between plasma oxidative balance and annual population change in *Pygoscelis* penguins

Model	OXY	d-ROM
Model 1: GLM		
Fixed factor: colony	*F*_4,99_ = 14.94, *P* < 0.001	*F*_4,99_ = 35.68, *P* < 0.001
Model 2: GLMM		
Covariate: annual population change	*F*_1,3_ = 26.84, *P* = 0.012	*F*_1,3_ = 2.24, *P* = 0.23
Model 3: GLMM		
Covariate: annual population change	*F*_1,3_ = 28.92, *P* = 0.009	*F*_1,3_ = 1.74, *P* = 0.28
Covariate: d-ROM	*F*_1,17_ = 2.82, *P* = 0.11	—
Covariate: OXY	—	*F*_1,98_ = 1.84, *P* = 0.18

Abbreviations: d-ROM, oxidative damage; and OXY, antioxidant capacity. The d-ROM data were ln transformed for normality. We checked for potential effects of the date of sampling and the sex of penguins on oxidative balance but did not find any (all *P* > 0.13). Consequently, we did not include these parameters in final models. In Model 1 (general linear model; GLM), we compared oxidative balance between the five considered colonies (Table 1). in order to assess if, irrespective of location and species, annual oxidative balance was related to the population trend of *Pygoscelis* penguins, we carried out Model 2 (general linear mixed model; GLMM), with OXY or d-ROM as dependent factors and annual population change as covariate. In this model, species was nested in location (as both Gentoo and Adélie penguins were sampled at Admiralty Bay) and used as a random factor. Finally, in Model 3, we repeated Model 2 but with d-ROM or OXY as covariate. Analyses were performed in SPSS 17.00 (SPSS Inc., Chicago, IL, USA).

Given that low oxidative damage may confer the same advantages in terms of fertility and survival as high antioxidant capacity (Helfenstein *et al.*, 2010; Freeman-Gallant *et al.*, 2011; Noguera *et al.*, 2012), lower levels of oxidative damage were also expected in increasing populations. However, even though oxidative damage also differed between colonies (Table 2, Model 1; Fig. 1), there was no general trend between oxidative damage and population trend (Fig. 2). This absence of relationship was confirmed when the species, the location, or the antioxidant capacity were taken into account in statistical analyses (Table 2, Models 2 and 3). The absence of relationship between oxidative damage and population trend may come from the fact that we sampled breeding individuals. Indeed, low levels of oxidative damage, expected in increasing populations, may be counterbalanced by elevated oxidative damage related to high investment in reproduction (Fletcher *et al.*, in press; Stier *et al.*, 2012), which is also expected in increasing populations, hence hiding any visible relationship between oxidative damage and population trend. In this context, it would be theoretically interesting to examine whether a negative relationship between oxidative damage and population trend is observed in penguins outside of the breeding season [which may be practically difficult because (i) birds are generally not present on the colony at that time, and (ii) population trend is generally calculated during breeding].

## Future directions and potential applications to conservation

The aim of this article is to stimulate conservation ecologists to integrate markers of oxidative balance into the array of physiological parameters used to monitor the health of animal populations in their natural habitat (Wikelski and Cooke, 2006). Recently published articles have provided solid evidence that variation in oxidative balance of free-ranging animals affects fitness components (Bize *et al.*, 2008; Freeman-Gallant *et al.*, 2011; Saino *et al.*, 2011; Losdat *et al.*, 2012; Noguera *et al.*, 2012). Here, by measuring the oxidative balance of *Pygoscelis* penguins from colonies with contrasting demographic trends, we show that variation in oxidative balance may be related to demographic processes, because we found a strong association between plasma antioxidant defences and population trends. These results suggest that antioxidant defences could be used by conservationists as an indicator to estimate the health of populations of unknown demographic trend. For instance, Gentoo penguins from O'Higgins Base (Antarctic Peninsula, 69°19'S, 57°53'W) have an antioxidant capacity of 253.1 ± 8.0 mmol^−1^ HOCl neutralized (*n* = 17, M. Beaulieu, personal data), but their population trend is unknown. Based on the results of our study (Fig. 2), this population is likely to be stable or slightly increasing.

As oxidative balance potentially shapes life-history trade-offs (Monaghan *et al.*, 2009), animal species are likely to modulate their oxidative balance with respect to their own life-history traits. For instance, experimentally increased breeding constraints result in opposite effects on the antioxidant defenses of Adélie penguins and zebra finches (*Taeniopygia guttata*), which show increased and decreased antioxidant capacity, respectively (Alonso-Álvarez *et al.*, 2004; Wiersma *et al.*, 2004; Beaulieu *et al.*, 2011). This discrepancy may be explained by the fact that Adélie penguins are long lived and favour self-maintenance, while zebra finches are short lived and favour current reproduction at the expense of self-maintenance (Stearns, 1989). It is therefore possible that a study examining the relationship between oxidative balance and population trend in a short-lived species would show opposite results to those obtained in long-lived *Pygoscelis* penguins. In that case, increasing populations of zebra finches would have lower antioxidant defences during reproduction than declining populations. This example also emphasizes the importance of considering the life stage when measuring oxidative balance to assess population health. Indeed, because the regulation of oxidative balance must be critical in terms of fitness only during oxidatively challenging conditions, oxidative markers may reflect population health only during life stages associated with higher vulnerability to oxidative damage (e.g. growth, reproduction, and ageing) or in oxidatively challenging environmental conditions (e.g. radioactive, hot environment). It is therefore possible that the relationship between population trend and antioxidant capacity that we observed in breeding *Pygoscelis* penguins would disappear in non-breeding penguins. In contrast, a negative relationship between oxidative damage and population trend may be apparent only outside the breeding season (see above). These examples emphasize the necessity to conduct further studies examining the relationship between oxidative balance and population trends in various conditions, for conservationists to use oxidative markers as indicators of population health. We therefore urge other research teams to explore this relationship further (i) in biological systems with different life-history traits, (ii) during different life stages, and (iii) in variable environmental conditions.
